# Androgen receptor differentially regulates the proliferation of prostatic epithelial cells *in vitro* and *in vivo*

**DOI:** 10.18632/oncotarget.11879

**Published:** 2016-09-07

**Authors:** Shu Yang, Ming Jiang, Magdalena M. Grabowska, Jiahe Li, Zachary M. Connelly, Jianghong Zhang, Simon W. Hayward, Justin M. Cates, Guichun Han, Xiuping Yu

**Affiliations:** ^1^ Department of Biochemistry and Molecular Biology, LSU Health Sciences Center, Shreveport, LA, USA; ^2^ Laboratory of Nuclear Receptors and Cancer Research, Center for Basic Medical Research, Nantong University School of Medicine, Nantong, Jiangsu, China; ^3^ Institute of Medicine and Public Health, Division of Epidemiology, Department of Medicine, Vanderbilt-Ingram Cancer Center, Vanderbilt University Medical Center, Nashville, TN, USA; ^4^ Department of Urologic Surgery, Vanderbilt University Medical Center, Nashville, TN, USA; ^5^ Department of Surgery, NorthShore University HealthSystem Research Institute, Evanston, IL, USA; ^6^ Department of Pathology, Microbiology and Immunology, Vanderbilt University Medical Center, Nashville, TN, USA; ^7^ Women's Health Division, Michael E. DeBakey Institute, and Department of Physiology and Pharmacology, College of Veterinary Medicine and Biomedical Sciences, Texas A&M University, College Station, TX, USA

**Keywords:** prostate, androgen receptor

## Abstract

Androgens regulate the proliferation and differentiation of prostatic epithelial cells, including prostate cancer (PCa) cells in a context-dependent manner. Androgens and androgen receptor (AR) do not invariably promote cell proliferation; in the normal adult, endogenous stromal and epithelial AR activation maintains differentiation and inhibits organ growth. In the current study, we report that activation of AR differentially regulates the proliferation of human prostate epithelial progenitor cells, NHPrE1, *in vitro* and *in vivo*. Inducing AR signaling in NHPrE1 cells suppressed cell proliferation *in vitro*, concomitant with a reduction in MYC expression. However, ectopic expression of AR *in vivo* stimulated cell proliferation and induced development of invasive PCa in tissue recombinants consisting of NHPrE1/AR cells and rat urogenital mesenchymal (UGM) cells, engrafted under renal capsule of adult male athymic mice. Expression of MYC increased in the NHPrE1/AR recombinant tissues, in contrast to the reduction seen *in vitro*. The inhibitory effect of AR signaling on cell proliferation *in vitro* were reduced by co-culturing NHPrE1/AR epithelial cells with prostatic stromal cells. In conclusion, these studies revealed that AR signaling differentially regulates proliferation of human prostatic epithelia cells *in vitro* and *in vivo* through mechanisms involving stromal/epithelial interactions.

## INTRODUCTION

Androgen deprivation therapy is the gold standard treatment for advanced stage PCa [1]. Initially, PCa responds to the treatment, resulting in tumor regression. However, these tumors almost invariably progress to castration-resistant PCa (CRPCa) in which androgen ablation can no longer suppress disease progression [2]. Studies have identified several mechanisms that contribute to the development of CRPCa, including androgen receptor (AR) amplifications, AR mutations, AR activation by growth factors, constitutively active AR variants, and increased intra-prostatic androgen synthesis [2]. Although neuroendocrine differentiation and cancer stem cell pathways may bypass AR, alterations in the androgen signaling pathway are still considered a predominant factor in mediating the emergence of resistance to androgen deprivation therapy in PCa.

Although blocking AR signaling causes prostate tumors to shrink in PCa patients and animal models, it has long been recognized that androgen deprivation therapy fails to produce complete responses. One explanation for the incomplete regression may be the presence of distinct populations of prostatic cells that respond to androgenic stimulation anomalously. The prostate gland has an epithelial parenchyma surrounded by a fibromuscular stroma. The epithelial tissue is composed of flattened basal cells and tall columnar secretory luminal cells with occasional neuroendocrine cells [3]. AR is expressed in both the stromal and epithelial tissues although the distribution between cell types varies among species. In humans, AR is expressed in virtually all luminal, many basal epithelial cells, and many cells of the fibromuscular stroma. Castration results in a significant reduction in the total volume of the prostate. In rats, the initial target of androgen loss is the microvasculature immediately adjacent to the epithelial cells with loss of epithelium occurring subsequent to the loss of vasculature [4, 5]. Experimental models have demonstrated that epithelial apoptosis following castration is due to a failure of androgen to occupy stromal, but not epithelial, AR [6, 7]. Androgen ablation leads to a preferential loss of the luminal phenotype. It does not however lead to a complete regression of the gland, and mechanisms such as Wnt/ß-catenin signaling seem to play a protective role, maintaining the viability of some portion of the tissue [8]. This maintenance of tissue is important in seasonally breeding animals [9], but is problematic in the context of cancer therapy in humans, where it allows the preservation of cancer cells from androgen deprivation therapy.

Studies using *in vitro* cell culture methods have shown that AR signaling exerts mixed effects on the growth of cultured prostatic cells [10–12]. Some AR-expressing PCa cells (such as LNCaP [10]) depend on androgens for proliferation/survival. However, other PCa cell lines are insensitive to androgens or show growth inhibition responses upon androgen exposure. For example, proliferation of PC3 cells, an AR-negative PCa cell line, is inhibited by ectopic-expression of AR [13, 14]. Similarly, proliferation of ARCaP cells that express low levels of AR is inhibited by androgen treatment both *in vitro* and *in vivo* [11]. LNCaP 104-R2, a sub-line cells derived from LNCaP after long-term androgen deprivation [12], expresses increased levels of AR. Unlike their parental cell line, LNCaP, androgen treatment induces cell cycle arrest and suppresses the cell proliferation of LNCaP 104-R2 [12]. Additionally, several recent studies have characterized the role of AR by ectopically expressing AR in normal prostatic epithelial cells [15–17]. These studies have revealed that AR signaling induces luminal epithelial differentiation and suppresses proliferation of these cells. Although these studies have established the roles of AR in *in vitro* cultured prostatic cells, it is not yet clear whether inducing AR signaling produces similar proliferation-regulation *in vivo*.

In this study, we ectopically expressed AR in human prostatic epithelial progenitor NHPrE1 cells and used a unique tissue recombination technique to investigate the roles of AR signaling in modulating prostatic cell proliferation *in vitro* and *in vivo.* NHPrE1 is a cell line derived from normal human prostate epithelial cells; NHPrE1 cells have some progenitor features [18]. When recombined with inductive rat urogenital sinus mesenchyme (UGM), NHPrE1 cells are able to generate benign secretory ductal-acinar architecture *in viv*o [18]. Thus, the benign nature of NHPrE1 cells makes them a suitable model system for investigating the molecular mechanisms of human prostatic carcinogenesis.

## RESULTS

### Ectopic expression of AR confers a functional AR-mediated signaling in NHPrE1 cells

**Figure 1:**
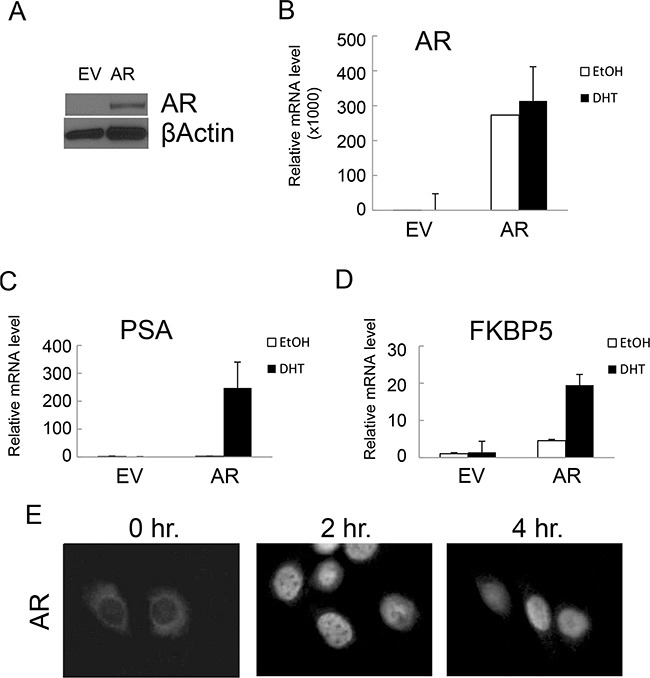
Ectopic expression of AR conferred functional AR-mediated androgen signaling in NHPrE1 cells Retroviral vector pLNCX or pLNCX-AR was used to generate NHPrE1 cells with empty vector (EV) control or AR transgene. **A.** Western blot to analyze the expression of AR in NHPrE1/EV (EV) or NHPrE1/AR (AR) cells. Beta-actin served as a loading control. **B-D.** quantitative (q)RT-PCR to assess the levels of AR (B) and androgen responsive genes PSA (C) and FKBP5 (D). Androgen treatment (DHT, 10 nM) induced the expression of PSA and FKBP5. The expression of GAPDH was used to normalize the qPCRs. **E.** immunofluorescence staining of AR. NHPrE1/AR cells were cultured in androgen-depleted medium for 24 hours and then treated with R1881 (1nM) for 2 or 4 hours. Immunofluorescence staining of AR was conducted to examine the nuclear translocation of AR upon androgen treatment.

NHPrE1 is an epithelial cell line derived from a normal human prostate that has some stem/progenitor features but does not express AR in 2D culture *in vitro*. When recombined with rat UGM and grafted *in vivo*, NHPrE1 cells form organized, functional prostatic glandular structures and therefore can be considered to represent untransformed prostate epithelium [18]. In order to study the role of AR in prostatic cells, we stably integrated full-length AR cDNA into NHPrE1 cells (NHPrE1/AR); NHPrE1 cells stably transduced with empty vector (EV) served as control cells. Ectopic expression of AR in NHPrE1/AR cells under the CMV promoter was confirmed by Western blot (Figure 1A) and quantitative (q)RT-PCR (Figure 1B). We also determined whether ectopic expression of AR enabled functional androgen signaling in NHPrE1 cells by examining the expression of two well-established androgen-regulated genes (PSA and FKBP5) by qRT-PCR. Results (Figures 1C and 1D) demonstrated that androgen treatment induced expression of both PSA and FKBP5 in NHPrE1/AR cells. Immunofluorescence staining for AR was conducted to examine cellular localization in response to androgens in NHPrE1/AR cells. Upon androgen treatment, ectopically expressed AR translocated from the cytoplasm to the cell nucleus (Figure 1E). These results confirm that ectopic expression of AR confers functional AR signaling in NHPrE1 cells.

### Inducing AR signaling inhibits the proliferation of NHPrE1 cells *in vitro*

**Figure 2:**
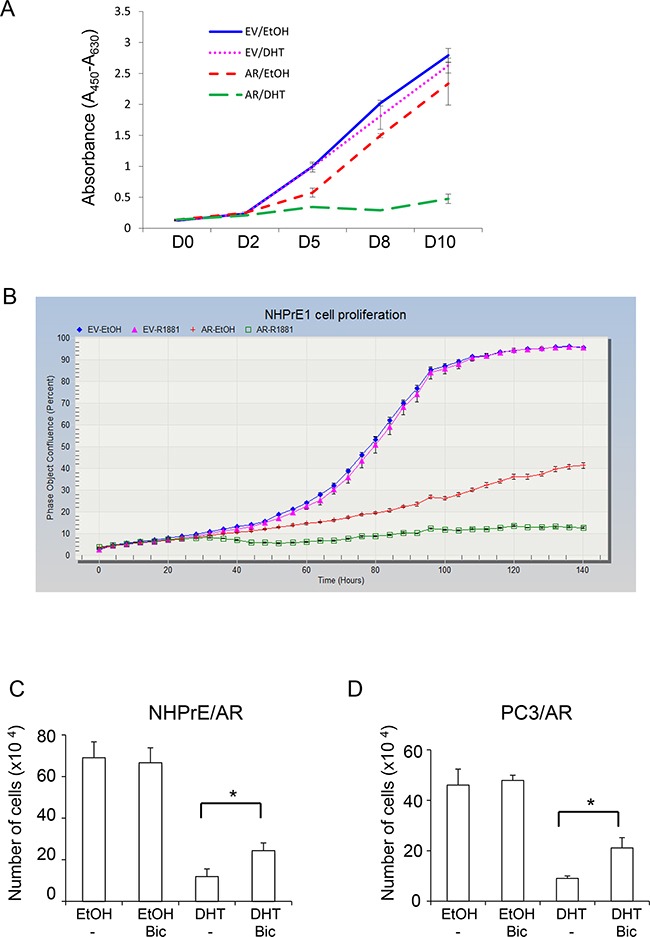
Androgen differentially regulated prostatic cell proliferation The proliferation of NHPrE1 cells with or without AR expression was assessed by using both WST-1 **A.** and IncuCyte **B.** methods. NHPrE1/EV cells and NHPrE1/AR cells were cultured in the absence or presence of androgens (10 nM DHT or 1 nM R1881). Androgen treatment had negligible effects on the proliferation of empty vector control cells, but suppressed proliferation of AR-expressing NHPrE1 cells (panel B, p<0.01 from 36 hour onward, comparison between ethanol- and R1881- treated NHPrE1/AR cells). Overall, compared with NHPrE1/EV cells, NHPrE1/AR cells displayed suppressed cell proliferation. **C** and **D**, blocking AR attenuated androgen-induced proliferation inhibition. NHPrE1/AR (C) or PC3/AR cells (D) were cultured with or without androgen (10 nM DHT) in the presence or absence of 10 μM bicalutamide (Bic) for 5 days. While DHT suppressed the proliferation of NHPrE1/AR and PC3/AR cells, addition of bicalutamide attenuated this inhibitory effect of androgens. *p<0.05, t-test.

To investigate whether ectopically expressed AR plays a functional role in modulating proliferation of NHPrE1 cells, we cultured NHPrE1/EV or NHPrE1/AR cells in medium supplemented with 5% charcoal-stripped serum with or without the addition of androgens (10 nM DHT or 1 nM R1881). As shown in Figure 2A (WST-1 assay) and Figure 2B (IncuCyte cell proliferation assay), androgen treatment did not affect proliferation of NHPrE1/EV cells but markedly inhibited proliferation of NHPrE1/AR cells. It was also noticed that 70% NHPrE1/AR cells died after they were cultured in the presence of androgens for 4 days. Additionally, we treated NHPrE1/AR cells with an AR inhibitor (bicalutamide, 10 μM) [19]. As shown in Figure 2C, bicalutamide did not alter the proliferation of NHPrE1/AR cells in the absence of androgen, but partially restored proliferation of NHPrE1/AR cells in the presence of androgen (p<0.05). It has been well documented that inducing AR signaling inhibits the proliferation of PC3/AR cells [13, 14, 20–22]. In our study, treatment of PC3/AR cells with bicalutamide also induced a growth restoration effect (Figure 2D). Together, these results suggest that inhibition of cell proliferation by androgen signaling in NHPrE1/AR cells is mediated by AR.

### Androgen differentially regulates MYC levels in prostatic cells

**Figure 3:**
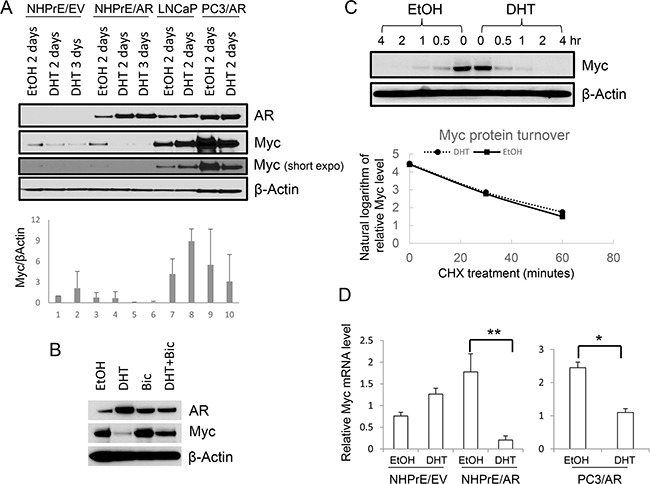
Androgen differentially regulated MYC expression **A.** Western blot for AR and MYC in prostatic cells. Androgen (DHT, 10 nM) treatment resulted in up-regulation of MYC in LNCaP cells, but down-regulation of MYC in NHPrE1/AR and PC3/AR cells. Lower panel is the quantification of MYC Western blot. **B.** Western blot for AR and MYC. NHPrE1/AR cells were cultured in androgen-depleted medium for 2 days with or without the addition of 10 nM DHT and/or 10 μM Bicalutamide (Bic). Bicalutamide treatment reversed androgen-mediated reduction of MYC. **C.** Analysis of MYC protein stability. Cycloheximide chase analyses were conducted using NHPrE1/AR cells to determine whether androgen treatment affected the turnover of MYC. NHPrE1/AR cells were treated with 50 μg/ml cycloheximide to block protein synthesis in the presence or absence of 10 nM DHT and harvested at different time points post treatment. Androgen treatment did not alter the stability of MYC protein in NHPrE1/AR cells. Lower panel is the semi-logarithm plot of MYC levels at different times of cycloheximide treatment. **D.** qRT-PCR to assess the levels of MYC mRNA in NHPrE1/EV, NHPrE1/AR, and PC3/AR cells. The expression of GAPDH was used to normalize the qPCRs. DHT treatment significantly decreased the level of MYC mRNA in NHPrE1/AR and PC3/AR cells. *p<0.05, **p<0.01, t-test.

MYC is a nuclear protein that plays important roles in cell cycle regulation. MYC is often amplified and/or mutated in cancer, especially in the prostate where it can play a role as an oncogene [23, 24]. Studies indicate that MYC is implicated in AR-mediated growth modulation of prostatic cells [12, 15, 16]. To determine whether MYC is also involved in the function of ectopically expressed AR in NHPrE1 cells, we examined the levels of MYC in NHPrE1/EV and NHPrE1/AR cells under DHT treatment. LNCaP and PC3/AR cells were used as controls. The results (Figure 3A) showed that levels of MYC were associated with androgenic modulation of proliferation of NHPrE1, LNCaP, and PC3 cells. DHT (10 nM) treatment resulted in a down-regulation of MYC in NHPrE1/AR as well as PC3/AR cells, correlating with the inhibitory effects of androgens in both cell types (Figure 2A and reference [13]). In contrast, DHT induced an up-regulation of MYC in LNCaP cells, a well-established androgen-dependent PCa cell line [10]. We also examined whether bicalutamide treatment could reverse androgen-mediated down-regulation of MYC in NHPrE1/AR cells. As shown in Figure 3B, DHT treatment caused a reduction of MYC in NHPrE1/AR cells, but the addition of bicalutamide restored MYC expression in NHPrE1/AR cells. In summary, the expression of MYC is associated with AR-mediated growth-inhibition of NHPrE1 cells.

Proteasomal degradation is one of the key mechanisms that regulates intracellular MYC levels [25]. To determine whether androgen treatment affects the stability of MYC protein, cycloheximide chase analyses were conducted using NHPrE1/AR cells cultured with or without androgen. The results showed that androgen treatment did not affect the turnover of MYC protein in these cells as MYC protein in NHPrE1/AR cells displayed a similar degradation pattern regardless of whether DHT was present or not (Figure 3C). Also, we assessed whether AR signaling affected the levels of MYC mRNA in these cells. As shown in Figure 3D, DHT treatment caused a significant reduction of MYC mRNA in both NHPrE1/AR and PC3/AR cells (p<0.01 and 0.05, respectively). Together, these results suggest that inducing AR signaling in NHPrE1 cells down regulates MYC mRNA level and subsequently decreases MYC protein expression, but does not alter proteasomal degradation of MYC.

### Ectopic expression of AR promotes NHPrE1 cells to form invasive PCa *in vivo*

**Figure 4:**
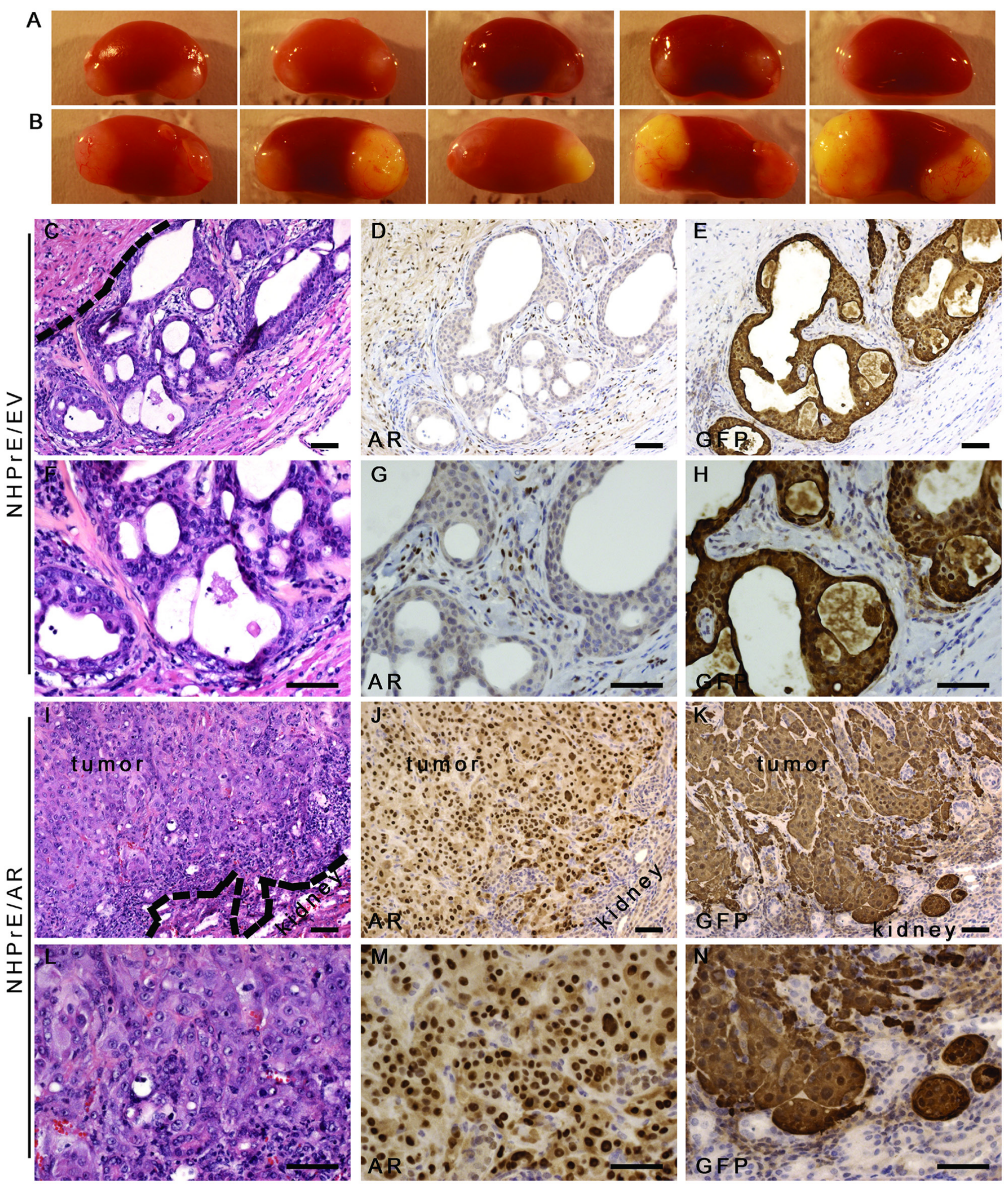
Ectopic-expression of AR transformed NHPrE1 cells *in vivo* NHPrE1/EV or NHPrE1/AR cells were recombined with rat UGM and grafted *in vivo*. **A** and **B.** gross morphology of renal subcapsular grafts. A, grafts derived from empty vector control NHPrE1/EV cells showed limited growth; B, grafts derived from NHPrE1/AR cells grew extensively. **C-N.** H&E and IHC staining performed on serial sections derived from NHPrE1/EV (C-H) or NHPrE1/AR (I-N) grafts. F-H and L-N are higher magnification pictures of C-E and I-K, respectively. Broken lines in panels C and I indicate the interface between the NHPrE1 grafts and host kidneys. While a clear boundary existed between the NHPrE1/EV graft and host kidney (C), NHPrE1/AR tumors focally invaded renal parenchyma (I-K). While epithelial cells in NHPrE1/AR grafts were positive for AR by IHC staining (J and M), epithelial cells in NHPrE1/EV graft showed little AR immunoreactivity (D and G). Stromal cells in NHPrE1/EV grafts (derived from rat UGM) were positive for AR staining (D and G). Epithelial cells in NHPrE1/EV grafts showed positive IHC staining for GFP and formed glandular structures (E and H); whereas GFP-tagged NHPrE1/AR cells (K and N) formed invasive carcinomas. Scale bars represent 25 μm.

In order to study how ectopic expression of AR modulates the proliferation of NHPrE1 cells *in vivo*, we conducted tissue recombination-xenografting experiments. Epithelial cells were combined with prostate-inductive mesenchymal cells, grafted under the renal capsules of immune-deficient male mice and allowed to grow for 3 months [26]. We used rat urogenital sinus mesenchyme (UGM), which can induce some prostatic epithelial cells to form prostatic glandular structures [18, 27]. For the epithelia, we used NHPrE1/EV control cells and NHPrE1/AR cells. Previous research has shown that when recombined with UGM and grafted *in vivo,* NHPrE1 cells form glandular structures [18], thereby allowing us to study how ectopic expression of AR alters the cell behavior *in vivo* and how signals from prostatic stromal cells regulate the proliferation of NHPrE1 cells through stromal/epithelial interactions. Our results showed that while the growth of NHPrE1/EV grafts was grossly negligible (Figure 4A), NHPrE1/AR grafts formed large invasive tumors (Figure 4B). To trace the epithelial cells in the NHPrE1/UGM tissue recombinants, we used immunohistochemical staining for GFP that was also expressed in these cells. We confirmed that the epithelial cells in the grafts were indeed NHPrE1 cells and were not contaminated with rat urogenital sinus epithelial cells. As shown in Figures 4C-4N, GFP-positive cells were detected in one of ten NHPrE1/EV grafts (Figures 4E and 4H), and the histology of this graft showed prostate glandular structure (Figures 4C and 4F). In contrast, eight of ten NHPrE1/AR grafts showed positive GFP IHC staining (Figures 4K and 4N). The inductive UGM dictated NHPrE1/EV cells to form benign glandular structures (Figures 4C and 4F), whereas the NHPrE1/AR recombinants developed invasive carcinomas (Figures 4I and 4L). No distant metastases were observed in any graft-bearing mice.

Although previous studies have indicated that tissue recombinants derived from early passages of NHPrE1 cells showed mature glandular differentiation with positive staining for AR in the glandular epithelial cells [18], less complete differentiation within luminal epithelium that was not clearly tall columnar and more limited AR expression was observed in the epithelial cells of the NHPrE1/EV grafts (Figures 4D and 4G). As expected, UGM-derived stromal cells were positive for AR. In contrast to the limited epithelial AR expression in NHPrE1/EV grafts, grafts derived from NHPrE1/AR cells showed strong AR staining in epithelial cells (Figures 4J and 4M), indicating that these cells did not lose the AR transgene during the three month *in vivo* growth phase without drug selection pressure.

**Figure 5:**
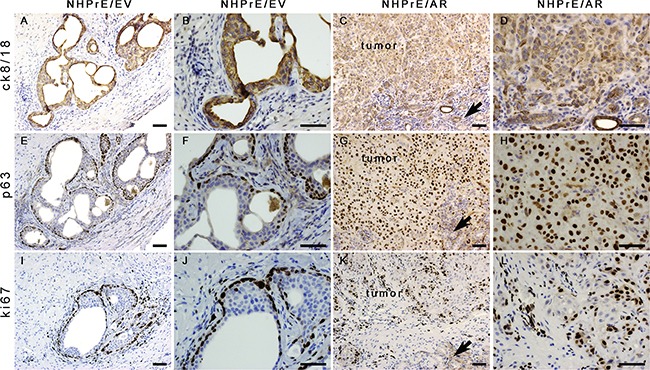
Histology of NHPrE1/EV and NHPrE1/AR grafts IHC stains for cytokeratin 8/18 (ck8/18, luminal epithelial cell marker), p63 (basal epithelial cell marker), and ki67 (cell proliferation marker) were performed on serial sections derived from NHPrE1/EV **A, B, E, F, I**, and **J.** or NHPrE1/AR grafts **C, D, G, H, K**, and **L.** B, D, F, H, J, and L are higher magnification photomicrographs of A, C, E, G, I, and K, respectively. Arrows in panels C, G, and K indicate host kidney. While NHPrE1/EV control cells formed glandular structures consisting of cytokeratin 8/18-positive luminal epithelial cells (A and B) and p63-positive basal cells (E and F), NHPrE1/AR cells formed invasive carcinomas that were positive for both cytokeratin 8/18 (C and D) and p63 IHC staining (G and H). Ki67 was barely detectable in luminal epithelial cells from NHPrE1/EV grafts (I and J), but was present in many basal cell nuclei in NHPrE1/EV grafts (I and J), as well as in malignant cells in NHPrE1/AR grafts (K and L). Scale bars represent 25 μm.

In the one NHPrE1/EV graft that grew, epithelial cells formed pseudostratified glandular structures consisting of cytokeratin 8/18-positive luminal epithelial cells (Figures 5A and 5B) and p63-positive basal cells (Figures 5E and 5F). In contrast, the invasive carcinomas formed by the NHPrE1/AR grafts were weakly positive for cytokeratin 8/18 (Figures 5C and 5D) and strongly positive for p63, a prostate basal cell marker (Figures 5G and 5H). A high proportion of malignant cells in the NHPrE1/AR grafts showed nuclear immunoreactivity for the cell proliferation marker Ki67 (Figures 5K and 5L), but only a few positive nuclei were seen in the stratified luminal epithelial cells from NHPrE1/EV grafts (Figures 5I and 5J). Interestingly, most basal cells of the NHPrE1/EV graft were positive for Ki67 (Figures 5I and 5J). Overall, more Ki67 positive cells (including both luminal and basal epithelium) were detected in NHPrE1/AR than NHPrE1/EV grafts (Table 1). Taken together, these results indicate that ectopic expression of AR promotes NHPrE1cells to form invasive PCa *in vivo*.

**Table 1:** Quantification of immunostaining

	Ki67 % Mean (SD)	Myc % Mean (SD)	pSTAT3 % Mean (SD)
NHPrE1/EV	18.3	9.8	4.4
NHPrE1/AR	39.8 (5.3)	40.6 (2.6)	36.8 (15.8)

### Expression of MYC and pSTAT3, but not FOXA1, is induced in NHPrE1/AR grafts

**Figure 6:**
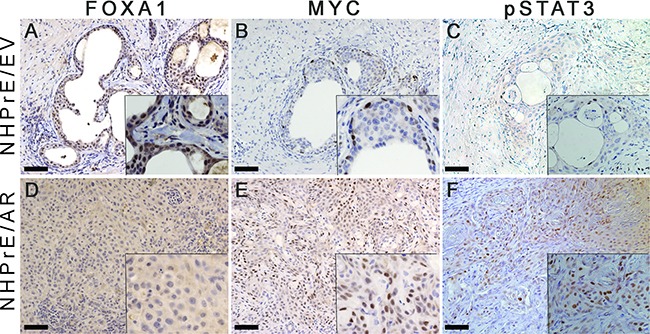
Expression of MYC and pSTAT3, but not FOXA1 was increases in NHPrE1/AR grafts IHC staining for FOXA1, MYC, and pSTAT3 was performed on serial sections derived from NHPrE1/EV and NHPrE1/AR grafts. Insets in each panel are higher magnification photomicrographs. While NHPrE1/EV grafts showed weak IHC staining for FOXA1 **A.** FOXA1 was not expressed in the majority of epithelial cells in NHPrE1/AR grafts **D.** While only a few basal cells in NHPrE/EV grafts displayed immunoreactivity MYC **B.** MYC was highly expressed in tumor cells from NHPrE1/AR grafts **E.** Malignant cells in some areas of NHPrE1/AR grafts were also positive for pSTAT3 **F.** whereas pSTAT3 levels were negligible in epithelial cells of NHPrE1/EV grafts **C.**

Previous research has shown that AR partners with various transcription factors to regulate distinct sets of genes involved in modulating the differentiation and proliferation of prostatic cells [28]. In an effort to explore the mechanisms that transform NHPrE1/AR cells to form invasive cancers *in vivo*, we examined the expression of FOXA1, a well-established AR co-activator [29], as well as MYC and pSTAT3, two genes that are differentially recruited to the AR transcriptome [28], in tissue recombinants derived from NHPrE1/EV and NHPrE1/AR cells. We showed that FOXA1 was weakly expressed in a subpopulation of glandular epithelial cells within the NHPrE1/EV graft (Figure 6A). However, little or no FOXA1 expression was detected in the malignant cells of NHPrE1/AR grafts (Figure 6D), indicating a lack of induction of FOXA1 by stromal signals in NHPrE1/AR cells.

Our *in vitro* study indicated that expression of MYC was directly associated with proliferation of NHPrE1 cells. To study whether MYC is associated with tumorigenicity of NHPrE1 cells in *vivo*, we used IHC staining to assess the expression of MYC in grafts derived from NHPrE1/AR or empty vector control NHPrE1/EV cells. Our results showed that while MYC was only expressed in a few basal cells in NHPrE1/EV grafts (Figure 6B), many more MYC-positive cells were detected in the carcinomas that formed in NHPrE1/AR grafts (Figure 6E and Table 1). These data indicate that AR regulates MYC expression in NHPrE1/AR cells in a context-dependent manner: suppressing MYC expression and inhibiting cell proliferation in 2D *in vitro* culture, but elevating MYC expression and promoting carcinoma formation *in vivo*.

A possible explanation for the incongruous regulation of NHPrE1 proliferation by AR signaling *in vitro* and *in vivo* is the presence of stromal/epithelial communication within tissue recombinants. Since signal transducer and activator of transcription 3 (STAT3) is instrumental in several signaling pathways that mediate prostatic stromal/epithelial cell interactions [30], we examined activated pSTAT3 (Tyr-705) expression in grafts derived from NHPrE1/EV and NHPrE1/AR cells. As shown in Figures 6C & 6F, pSTAT3 is barely detectable in the epithelial cells of empty vector control grafts but numerous pSTAT3-positive cells were observed in NHPrE1/AR grafts (Figures 6C & 6F and Table 1), indicative of active STAT3 signaling in these grafts.

### The presence of stromal cells restores proliferation of NHPrE1/AR cells

**Figure 7:**
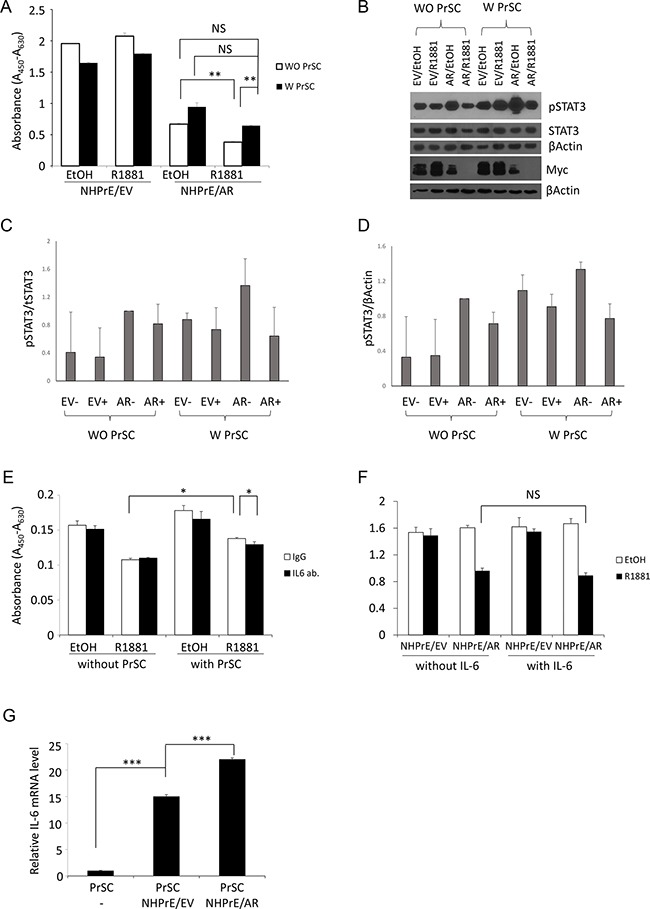
Stromal/epithelial interactions are involved in modulating the proliferation of NHPrE1 cells **A.** WST-1 cell proliferation assay to assess proliferation of NHPrE1 cells in the presence or absence of prostatic stromal cells (PrSC). NHPrE1/EV and NHPrE1/AR cells were co-cultured with PrSC for 5 days. While androgen treatment (1 nM R1881) inhibited proliferation of NHPrE1/AR cells in the absence of PrSC, co-culture with PrSC stimulated proliferation of NHPrE1/AR cells and partially reversed the proliferation inhibitory effect of androgens seen *in vitro*. ** p<0.01, t-test. **B.** Western blots to assess levels of pSTAT3 in NHPrE1/EV or NHPrE1/AR cells cultured with or without PrSC for 2 days. Levels of pSTAT3 (Tyr-705) and total STAT3 in NHPrE1/EV and NHPrE1/AR cells were compared; beta-Actin served as loading control. Co-culture with PrSC increased the levels of pSTAT3 but not MYC in NHPrE1 cells. **C** and **D.** quantification of pSTAT3 Western blot. The levels of pSTAT3 were normalized by total STAT3 (C) or by beta-Actin (D). **E.** blocking IL-6 attenuated the stimulatory effect of PrSC on NHPrE1 cell proliferation. NHPrE1/AR were cultured with or without PrSC cells in the presence or absence of IL-6 neutralizing antibody for 3 days. Anti-IL-6 attenuated the proliferation stimulation effect of PrSC. * p<0.05, t-test. Similar trend was observed in additional independent experiments. **F.** WST-1 cell proliferation assay. NHPrE1 cells were cultured in the presence or absence of androgens (1 nM R1881) with or without the addition of IL-6 (25 ng/ml). Addition of IL-6 to the cell culture medium did not induce the proliferation of NHPrE1 cells. **G.** RT-qPCR to assess expression of IL-6 in PrSC cells. Prostate stromal cells (PrSC) were cultured in the presence or absence of NHPrE1/EV (N/EV) or NHPrE1/AR (N/AR) cells. Co-culture with prostate epithelial cells stimulated production of IL-6 mRNA in PrSC cells. ***p<0.001, t-test.

**Figure 8:**
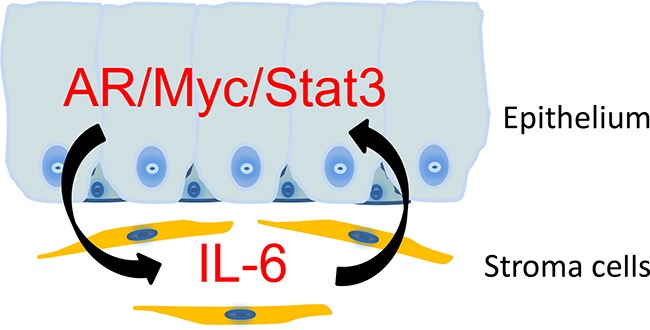
A schematic representation on the stromal/epithelial interaction in NHPrE1/AR tissue recombinants NHPrE1 cells produce factors that induce the expression of IL-6 in prostate stroma. Prostate stromal cells promote the proliferation of NHPrE1 cells via a mechanism that involves the induction of MYC and pSTAT3 in NHPrE1/AR cells. The combined expression of AR, MYC, and pSTAT3 may transform NHPrE1 cells *in vivo*.

To determine the role of stromal cells in regulating the proliferation of NHPrE1 cells, stromal/epithelial co-culture experiments were conducted. The results showed that the presence of prostate stromal cells (PrSC) promoted the proliferation of NHPrE1/AR cells. Specifically, when NHPrE1/AR cells were co-cultured with PrSC cells, the inhibitory effect of androgens on cell proliferation was diminished (Figure 7A). These results suggest that factors secreted from prostatic stromal cells may stimulate proliferation of NHPrE1/AR cells. Given that pSTAT3 was induced in the NHPrE1/AR tissue recombinants, we hypothesized that the IL-6/STAT3 pathway, a well-established mechanism of stromal/epithelial communication [30], was involved in the crosstalk between NHPrE1/AR and stromal cells. To test this hypothesis, we first assessed the levels of pSTAT3 (Tyr-705) in NHPrE1/EV and NHPrE1/AR cells cultured in the presence or absence of stromal cells. The results showed that co-culture with PrSC cells increased the levels of pSTAT3 in both NHPrE1/EV and NHPrE1/AR cells (Figures 7B-7D). However, co-culture with PrSC did not induce the expression of MYC in these cells (Figure 7B), suggesting that elevated MYC in NHPrE1/AR tissue recombinants may result from the presence of other cellular components in the tumor microenvironment. Also, we utilized an IL-6 neutralizing antibody to block IL-6 signaling in the co-culture system. The IL-6 levels in the cell culture supernatant were measured by ELISA to confirm that addition of IL-6 neutralizing antibody effectively decreased IL-6 levels in the cell culture media (Table 2). While co-culture with stromal cells promoted proliferation of NHPrE1/AR cells, concomitant with an increase of IL-6 in cell co-culture media, addition of IL-6 neutralizing antibodies decreased IL-6 level in cell co-culture media and partially attenuated the restoration of cell proliferation induced by stromal cells (Figure 7E, p<0.05). These results suggest that IL-6 pathway is involved in PrSC/NHPrE1 communications. However, addition of IL-6 (25 ng/ml) to the cell culture medium failed to induce the cell proliferation of NHPrE1 cells (Figure 7F), indicating that IL-6 alone is not sufficient to promote the proliferation of NHPrE1/AR cells and additional signals from PrSC are indispensable for stimulating the proliferation of these cells. Further, we analyzed the relative levels of IL-6 mRNA in PrSC cells, as well as in PrSC cells co-cultured with NHPrE1/EV or NHPrE1/AR cells (Figure 7G). The results showed that when co-cultured with epithelia, PrSC cells produced more IL-6 than when cultured alone (p<0.001). A schematic illustration on the two-way stromal/epithelial communication was summarized in Figure 8. Together, these data suggest that stromal cells may help to restore proliferation of NHPrE1/AR cells by releasing IL-6 and possibly other pro-growth factors.

**Table 2:** IL-6 concentration in the culture media of NHPrE1/AR cells

	Secreted IL-6 (pg/ml)			
	Without PrSC		With PrSC	
	EtOH Mean (SD)	R1881 Mean (SD)	EtOH Mean (SD)	R1881 Mean (SD)
IgG (Control)	226.8 (7.7)	100.0 (6.9)	537.7 (1.0)	298.5 (1.5)
IL-6 Blocking Antibody	2.3 (0.2)	2.6 (0.2)	2.6 (0.2)	2.6 (0.2)

## DISCUSSION

Although several studies have examined the growth-modulating effects of inducing AR signaling in prostatic cells *in vitro*, the *in vivo* effect of ectopic-expression of AR has not been well defined. In this study, we chose NHPrE1 cells as a model system to ectopically express AR and study the *in vitro* and *in vivo* effects of inducing AR signaling in these cells. The capacity of NHPrE1cells to form glandular structures when recombined with inductive UGM enabled us to investigate how ectopic expression of AR changed prostatic histomorphology. We found that inducing AR signaling inhibited the proliferation of NHPrE1cells *in vitro*, but surprisingly promoted NHPrE1 cells to form invasive tumors *in vivo*.

In an effort to decipher the mechanisms that caused the differential proliferative responses of NHPrE1 cells to AR signaling *in vitro* and *in vivo*, we conducted stromal/epithelial cell co-cultures. Prostatic fibroblasts, the major cellular components of the tumor microenvironment, were used in our co-culture study. Other cellular components, such as endothelium and immune cells, have yet been tested. Our results showed that the presence of prostate stromal cells (PrSC) diminishes the inhibitory effects of androgen, suggesting that factors secreted from PrSC stimulate proliferation of NHPrE1/AR cells. Furthermore, we explored the signaling pathways that might mediate this stromal/epithelial interaction and found that blocking IL-6 signaling partially attenuated the growth restoration effect of stromal cells, indicating that IL-6/STAT3 is one of the mechanisms through which PrSC and NHPrE1/AR cells communicate. However, it was also noticed that blocking IL-6 led to only a small decrease in cell proliferation, indicating that other pathways are likely involved in the stroma and epithelia communication. Although co-culture with PrSC induced the level of pSTAT3 in NHPrE1 cells, the expression of MYC in these cells was hardly affected. This suggests that elevated MYC expression in the NHPrE1/AR tissue recombinants may result from signals from other cellular components, such as immune cells, of the tumor microenvironment. Our results also showed that prostate stromal cells expressed more IL-6 mRNA when they were co-cultured with NHPrE1 cells than when cultured alone, indicating that factors secreted from epithelial cells modulate gene expression in adjacent stromal cells. Further research is warranted to identify the factors that mediate the crosstalk from NHPrE1 to PrSC cells.

We found that inducing AR signaling inhibited the proliferation of NHPrE1 cells *in vitro*. This observation differs from a recent study in which androgens slightly promote the proliferation of NHPrE1 cells that have AR stably expressed [31]. We note that while the parental cells used in these two studies are the same, the generation of the AR expressing variants was performed separately with the CMV promoter driving expression in the cells used here and the EF1A promoter in the study reported by Austin et al. As a result these cell strains are not identical with different AR integration sites in the two NHPrE1-AR derivatives. This, along with the different gene expression levels elicited by the two promoters and somewhat different timing and culture conditions may explain the discrepancy observed in these studies.

AR expression was induced less strongly in epithelial cells of the NHPrE1/EV + rUGM tissue recombinants described here than was expected based upon previous studies [18]. The previous study was performed using castrated SCID mice pelleted with testosterone [18], whereas intact nude mice were used in this current study. The lower testosterone levels in the host mice may explain the lower level of induction of AR in the NHPrE1/EV control graft and the less complete differentiation of the epithelial structures illustrated here. We also found that ectopic expression of AR promoted NHPrE1cell proliferation when recombined with UGM and grafted *in vivo*. This result appears to be at odds with previous *in vivo* studies showing that knockout of AR in prostate luminal epithelial cells results in increased cell proliferation, suggestive of a growth-inhibitory effect of AR signaling in these cells [32]. One explanation for this discrepancy is that the luminal epithelial cells of AR knockout prostates are fully differentiated, but NHPrE1 cells have progenitor features [18]. While recombination with UGM can instruct NHPrE1/EV cells to form benign prostate glandular structures, constitutive expression of AR in NHPrE1 cells alters their response to gland-organizing signals from UGM, resulting in the development of carcinomas. It was also noteworthy that these tumors retained expression of the basal cell marker p63, perhaps suggesting that their pathogenesis is somewhat different from that of prostate tumors in the general population. Additionally, previous studies have shown that androgenic modulation of prostatic growth is biphasic, i.e., androgens either stimulate or inhibit proliferation of prostatic cells depending on the developmental stage of the organ [33, 34]. Whereas androgens stimulate prostatic growth in the prepubertal period, most prostatic cells enter proliferative quiescence after sexual maturation, despite the continuous presence of androgen. Accumulating evidence indicates that AR signaling provides a mechanism to suppress the proliferation of these fully differentiated prostatic luminal epithelial cells [32]. In line with this notion, ectopic expression of AR in PC3 cells induces differentiation [13] and suppresses the growth of PC3/AR tumors *in vivo* [22]. In our case, the progenitor features of NHPrE1 cells may enable these cells to escape from AR-mediated suppression of proliferation.

Our observation that AR signaling differentially regulates prostatic cell proliferation *in vitro* and *in vivo* is in line with a recent study conducted by Neal and colleagues that showed that AR induces a distinct transcriptional program *in vivo* that is not observed in cultured cells [28]. Using a ChIP-Seq approach, they showed that in cultured cells, AR binding sites are associated with potential FOXA1 and NFI binding sites to regulate a set of differentiation-related genes; whereas *in vivo*, AR potentially partners with MYC, STAT, and E2F to control the expression of a different set of genes that may modulate cell proliferation [28]. MYC is an oncogene frequently altered in advanced stage PCa [23, 24]. Over-expression of MYC confers an androgen-independent PCa cell growth *in* vitro [35], and prostate-specific over-expression of MYC results in the development of invasive PCa *in* vivo [36]. The association of MYC expression with AR modulation of the proliferation of NHPrE1 cells and the high expression of MYC in NHPrE1/AR tissue recombinants further suggests involvement of MYC in transforming NHPrE1/AR cells *in vivo*. IL-6/STAT3 is involved in the communications between prostate tumor cells and the microenvironment. Moreover, STAT3 is an important modulator of AR signaling in the prostate [30]. The combination of the expression of MYC, STAT3, and AR in NHPrE1/AR grafts may reprogram the AR transcriptome and promote neoplastic transformation of these cells.

Our study also found, in contrast to the elevated expression of MYC and pSTAT3, that FOXA1 was not expressed in the malignant cells in NHPrE1/AR grafts. However, FOXA1 expression was detected in the glandular epithelial cells of the NHPrE1/EV tissue recombinant, likely reflecting the differentiation status of the cells, illustrated by the absence of basal cell markers. FOXA1 is a well-established AR co-activator [29], and previous studies have suggested that the AR/FOXA1 complex is involved in controlling differentiation-related genes instead of proliferation-related genes in prostatic cells. For example, studies have shown that FOXA1 interacts with AR to regulate the expression of prostate-specific genes such as PSA, PAP, and SBP [29]. More recent studies have shown that, as a pioneer transcription factor, FOXA1 recruits AR to the promoters of a set of genes that define prostate specific differentiation. However, depletion of FOXA1 in PCa cells did not cause AR to lose all its binding sites; instead, the AR transcriptome was reprogrammed and new AR binding sites were found on the promoters of a distinct set of genes not observed in parental cells [37, 38]. These new AR target genes may be involved in promoting PCa progression. Consistent with the role of FOXA1 in regulating the differentiation of prostate epithelial cells, FOXA1 mutations are observed in advanced stage human PCa [39, 40] and inactivation of FOXA1 gene in prostate epithelial cells promotes prostatic hyperplasia in murine models [41]. Conversely, ectopic expression of FOXA1 inhibits the invasive capacity of PC3 and DU145 cells [40], thereby conferring a less aggressive phenotype in cells that represent advanced stage PCa. Taken together, these studies suggest that expression of FOXA1 restrains the AR transcriptome to genes related to differentiated function and that the lack of FOXA1 expression permits a switch in the AR transcriptome that results in enhanced cell proliferation. Therefore, in addition to the induction of MYC and pSTAT3, the lack of FOXA1 expression in NHPrE1/AR cells *in vivo* might be another contributor to, or indicator of, the transformation of these cells.

In conclusion, in this study, we found that AR signaling differentially regulated the proliferation of NHPrE1 cells *in vitro* and *in vivo* via mechanisms that involved prostate stromal/epithelial interactions.

## MATERIALS AND METHODS

### Cell culture

NHPrE1 cells [18] were maintained in DMEM (Gibco) containing 10% fetal bovine serum (FBS) (Atlanta Biologicals, Flowery Branch, GA), 1% penicillin/streptomycin (Gibco), 10 ng/ml epidermal growth factor (Sigma– Aldrich, St. Louis, MO), 1% insulin-transferrin-selenium (ITS) (Gibco), and 0.4% bovine pituitary extract (Atlanta Biologicals). NHPrE1 cells were transfected with EGFP-expressing plasmid; GFP-positive NHPrE1 cells were selected by cell sorting. To establish AR-expressing NHPrE1/GFP cells, CMV promoter-driven LNCX or LNCX-AR retroviral vector-based plasmids were transfected into Phoenix packaging cells (ATCC, Manassas, VA). Twenty-four hours later, culture media were collected and used to infect NHPrE1/GFP cells. The infection procedure was repeated twice. The transduced cells, stably expressing AR, were selected by culturing them in the presence of G418 (Sigma, St. Louis, MO, USA). G418 resistant cell populations were used in this study. American Type Culture Collection (ATCC) has authenticated NHPrE1 cells and no contamination from other type of cells was found. PC3/AR [21] and LNCaP (ATCC) cells were cultured in RPMI1640 supplemented with10% serum.

### Cell proliferation assay

Both IncuCyte and WST-1 methods were used to assess the proliferation of NHPrE1/EV and NHPrE1/AR cells. For IncuCyte cell proliferation assay, NHPrE1/EV or NHPrE1/AR cells (1500 cells per well, 96-well plate) were cultured in DMEM medium containing 5% charcoal-stripped, heat inactivated serum (Atlanta Biologicals) without other additives to avoid the cross-activation of AR by exogenous growth factors. After the cells are attached, the cell culture medium were changed to DMEM-5% charcoal-stripped serum with or without the addition of androgens (1 nM R1881 or 0.1% ethanol) for up to 140 hours. Cell confluence was monitored every 4 hours. For WST- assay (Roche Applied Science, Indianapolis, IN), NHPrE1/EV or NHPrE1/AR cells were cultured in DMEM-5% charcoal-stripped serum with or without the addition of 10 nM DHT. Cell culture medium were replenished daily when DHT was used due to its metabolic instability. WST-1 cell proliferation assay was conducted according to manufacturer's instruction. Ten μL WST-1 reagent was added to each well and incubated at 37°C for 1 hour. Absorbance at 440-450 nm (630 nm was used as reference wavelength) was measured using a microplate reader.

### Co-culture of prostate stromal and epithelial cells

NHPrE1 cells were cultured with or without primary prostate stromal cells (PrSC) (Lonza, Williamsport, PA). NHPrE1/EV (empty vector) or NHPrE1/AR cells were seeded overnight in 24-well plates in DMEM media containing 5% charcoal-stripped serum. The next day, a WST-1 assay was conducted to assess for equal seeding of each cell line. PrSC cells were then seeded into cell culture inserts (0.4 μm pores) and co-cultured with NHPrE1 cells in DMEM media containing 5% charcoal-stripped serum supplemented with R1881 (1 nM) (Sigma) or ethanol for 5 days. Cell culture medium were replenished every other day. In some experiments, IL-6 neutralizing antibody (final concentration 2 μg/ml, R&D system, Minneapolis, MN) was added to block IL-6 signaling; goat IgG served as a negative control. WST-1 cell proliferation assays were conducted to assess the proliferation of NHPrE1 cells that were co-cultured with PrSC according to the manufacturer's instructions. For experiments where the expression of IL-6 in PrSC cells was examined, PrSC cells were seeded in 6-well plates and co-cultured with NHPrE1/EV or NHPrE1/AR cells for 2 days. RNA was extracted from PrSC cells and levels of IL-6 mRNA were assessed by quantitative (q)RT-PCR.

### Western blot analysis

Protein lysates were prepared from prostatic cells as described previously [43]. Twenty-micrograms of total protein was loaded for electrophoresis. After transfer, membranes were blocked in 5% non-fat milk for one hour, incubated with primary antibodies at 4°C overnight, followed by incubation with horseradish peroxidase-conjugated secondary antibodies (GE Healthcare, Pittsburgh, PA) at room temperature for one hour. ECL-Plus detection system (PerkinElmer, Waltham, MA) was used to visualize immunolocalization. Rabbit antibodies against MYC were purchased from Epitomics (Burlingame, CA), AR from Santa Cruz Biotechnology (Santa Cruz, CA), STAT3 and pSTAT3 from Cell Signaling Technology (Danvers, MA), and β-actin from Sigma.

### Cycloheximide chase analysis

Cycloheximide chase analysis was conducted to determine the half-life of MYC in NHPrE1/AR cells in the presence or absence of androgen. Cells were treated with 50 μg/ml cycloheximide (Sigma) to block protein synthesis in the presence or absence of 10 nM DHT and harvested at 0, 0.5, 1, 2, and 4 hours post-treatment. Western blotting was performed using anti-MYC antibody and band intensities were measured by using Image J (NIH).

### Histology and immunohistochemical staining

Immunohistochemistry (IHC) was conducted as described previously [27]. Tissues were fixed in 10% buffered formalin overnight and processed to paraffin. IHC stains were performed following routine de-paraffinization and rehydration of 5 μm sections. Antigen retrieval was performed by microwaving slides for 20 min in boiling antigen-unmasking solution (Vector Laboratories, Burlingame, CA). Endogenous peroxidase activity was blocked with DAKO Peroxidase Blocking Reagent (DAKO, Carpinteria, CA) for 15 min. Sections were incubated with primary antibodies at 4°C overnight in a humidified chamber. Antibodies used were: AR, p63, FOXA1, and GFP (Santa Cruz Biotechnology), MYC (Epitomics, Burlingame, CA), pSTAT3 (Cell Signaling Technology), and Ki67 (Abcam, Cambridge, MA). Specific antibody binding was detected using the Vectastain Elite ABC peroxidase kit (Vector Laboratories, Burlingame, CA) according to the manufacturer's protocol with the DAKO DAB-Chromogen System (DAKO, Carpinteria, CA). Sections were counterstained with hematoxylin, dehydrated, and cover-slipped.

### Tissue recombination-Xenografting

Tissue recombination experiments were conducted as described previously [27]. Briefly, 6×10^5^ NHPrE1/EV or NHPrE1/AR cells were recombined with 3×10^5^ rat UGM cells in 50 μl of neutralized type I rat tail collagen to make the tissue recombinants. Solidified recombinants were cultured overnight and then grafted beneath the renal capsules of adult male nude mice. Host mice were sacrificed 3 months later by anesthetic overdose followed by cervical dislocation. Kidneys were excised, and grafts were dissected and processed for histology and immunohistochemistry. All the animal experiments were approved by the Institutional Animal Care and Use Committee.

### Statistical analysis

All the experiments were conducted in triplicate and repeated at least once. Statistical significance was evaluated using a two-sided Student's t test and a p-value of 0.05 was considered statistically significant. Quantification of immunohistochemistry staining was conducted using ImmunoRatio program.
